# (Dimethyl sulfoxide-κ*O*)[2-({(ethyl­sulfan­yl)[2-(2-oxidobenzyl­idene-κ*O*)hydrazin­yl­idene-κ*N* ^2^]meth­yl}­imino­meth­yl)­phenol­ato-κ*O*]dioxidouranium(VI)

**DOI:** 10.1107/S1600536812003789

**Published:** 2012-02-04

**Authors:** Reza Takjoo, Seik Weng Ng, Edward R. T. Tiekink

**Affiliations:** a Department of Chemistry, School of Sciences, Ferdowsi University of Mashhad, 91775-1436 Mashhad, Iran; b Department of Chemistry, University of Malaya, 50603 Kuala Lumpur, Malaysia; c Chemistry Department, Faculty of Science, King Abdulaziz University, PO Box 80203 Jeddah, Saudi Arabia

## Abstract

The U^VI^ atom in the title complex, [U(C_17_H_15_N_3_O_2_S)O_2_(C_2_H_6_OS)], exists within a distorted penta­gonal–pyramidal geometry where the oxide atoms occupy axial positions [O—U—O = 177.84 (14)°] and the penta­gonal plane is defined by the N_2_O_2_ atoms of the tetra­dentate Schiff base ligand and the O atom of the dimethyl sulfoxide mol­ecule. In the crystal, centrosymmetric aggregates are formed *via* pairs of C—H⋯O inter­actions. The azomethine C=N atoms and ethyl­thiolyl group are disordered over two orientations in a 0.828 (3):0.172 (3) ratio.

## Related literature

For background to uranyl Schiff base complexes, see: Şahin *et al.* (2010 ▶); Özdemir *et al.* (2011 ▶).
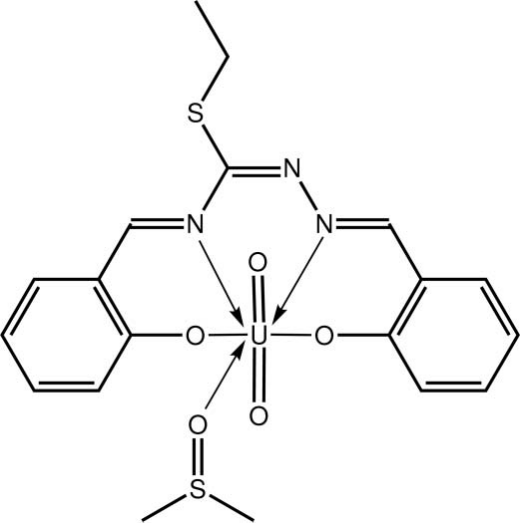


## Experimental

### 

#### Crystal data

[U(C_17_H_15_N_3_O_2_S)O_2_(C_2_H_6_OS)]
*M* *_r_* = 673.54
Monoclinic, 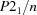

*a* = 11.6988 (3) Å
*b* = 15.4972 (3) Å
*c* = 12.2246 (3) Å
β = 105.714 (3)°
*V* = 2133.47 (9) Å^3^
*Z* = 4
Mo *K*α radiation
μ = 7.84 mm^−1^
*T* = 100 K
0.18 × 0.12 × 0.10 mm

#### Data collection

Agilent SuperNova Dual diffractometer with an Atlas detector
Absorption correction: multi-scan (*CrysAlis PRO*; Agilent, 2010 ▶) *T*_min_ = 0.333, *T*_max_ = 0.508
19265 measured reflections
4927 independent reflections
4237 reflections with *I* > 2σ(*I*)
*R*_int_ = 0.044

#### Refinement

*R*[*F* ^2^ > 2σ(*F* ^2^)] = 0.030
*wR*(*F* ^2^) = 0.063
*S* = 1.01
4927 reflections
281 parameters
3 restraints
H-atom parameters constrained
Δρ_max_ = 0.98 e Å^−3^
Δρ_min_ = −1.52 e Å^−3^

### 

Data collection: *CrysAlis PRO* (Agilent, 2010 ▶); cell refinement: *CrysAlis PRO*; data reduction: *CrysAlis PRO*; program(s) used to solve structure: *SHELXS97* (Sheldrick, 2008 ▶); program(s) used to refine structure: *SHELXL97* (Sheldrick, 2008 ▶); molecular graphics: *ORTEP-3* (Farrugia, 1997 ▶) and *DIAMOND* (Brandenburg, 2006 ▶); software used to prepare material for publication: *publCIF* (Westrip, 2010 ▶).

## Supplementary Material

Crystal structure: contains datablock(s) global, I. DOI: 10.1107/S1600536812003789/hb6614sup1.cif

Structure factors: contains datablock(s) I. DOI: 10.1107/S1600536812003789/hb6614Isup2.hkl

Additional supplementary materials:  crystallographic information; 3D view; checkCIF report

## Figures and Tables

**Table 1:** Selected bond lengths (Å)

U—O1	2.267 (3)
U—O2	2.233 (3)
U—O3	1.787 (3)
U—O4	1.792 (3)
U—O5	2.395 (3)
U—N1	2.547 (4)
U—N3	2.603 (4)

**Table 2:** Hydrogen-bond geometry (Å, °)

*D*—H⋯*A*	*D*—H	H⋯*A*	*D*⋯*A*	*D*—H⋯*A*
C5—H5⋯O4^i^	0.95	2.48	3.322 (5)	147

Symmetry code: (i) 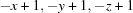.

**Fig. 1.**
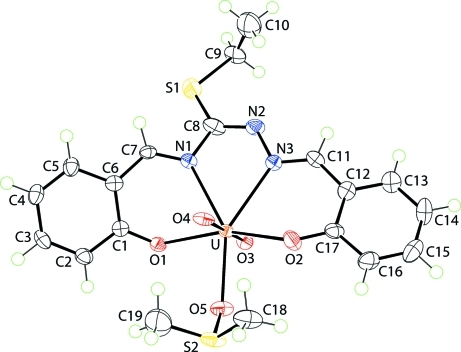
The molecular structure of (I) showing displacement ellipsoids at the 70% probability level. Only the major component of the disordered residue is shown.

**Fig. 2.**
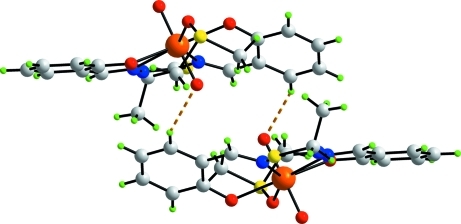
A view of the centrosymmetric aggregate in (I). The C—H···O interactions are shown as dashed lines.

**Fig. 3.**
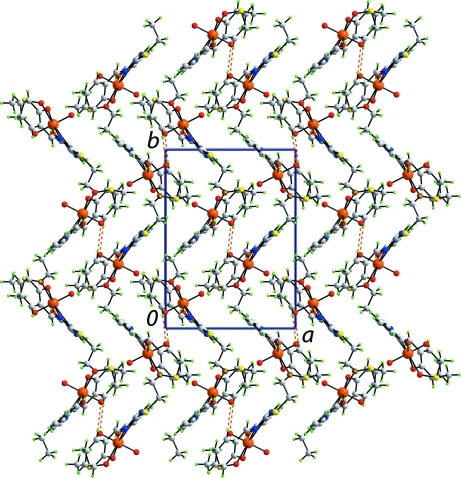
A view in projection down the c axis of the unit-cell contents of (I).

## supplementary crystallographic information

### Comment

Tetradentate ligands with N_2_O_2_ donor sets and their metal complexes are of great importance as they provide synthetic models for the metal-containing sites in metallo-proteins and metallo-enzymes, and display extensive catalytic and bioactive applications. Such considerations have motivated recent studies of uranyl Schiff base complexes (Şahin *et al.*, 2010; Özdemir *et al.*, 2011) and led to the synthesis of the title complex, (I).

The U atom in (I), Fig. 1, exists within a distorted pentagonal bipyramidal geometry with the axial positions occupied by the oxido-O atoms, O3—U—O4 = 177.84 (14)°. The pentagonal plane is defined by the N_2_O_2_ atoms, derived from the tetradentate Schiff base ligand, and the O atom of the dimethyl sulfoxide molecule, Table 1. The Schiff base ligand is somewhat buckled with the dihedral angle between the terminal benzene rings being 35.6 (2)°. The *S*-bound substituents are directed to one side of the molecule, Fig. 1.

In the crystal structure, centrosymmetric pairs of molecules are linked *via* C—H···O(oxido) interactions, Fig. 2 and Table 2. The dimeric aggregates stack into columns parallel to *c*, Fig. 3.

### Experimental

UO_2_(OAc)_2_.2H_2_O (0.42 g, 1.0 mmol) was added to an ethanol (20 cm^3^) solution of salicylaldehyde mono-*S*-ethylisothiosemicarbazone hydrobromide (0.32 g, 1.0 mmol) and salicylaldehyde (0.12 g, 1.0 mmol). The red solution was heated under reflux for 1 h at 70 °C. Red crystals of the product, (I), precipitated after three days, collected by filtration, washed with ethanol, and dried in air. Recrystallization was by slow evaporation (10 days) of a dimethyl sulfoxide solution of (I) which yielded red crystals. *M*.pt. 513 K. Yield: 46%.

### Refinement

Carbon-bound H-atoms were placed in calculated positions [C—H 0.95 to 0.99 Å, *U*_iso_(H) 1.2 to 1.5*U*_eq_(C)] and were included in the refinement in the riding model approximation. The ethylthiolyl unit is disordered over two positions; the minor component refined to a site occupancy = 0.172 (3). The *U*_iso_ parameters of the atoms of the minor component were constrained to be equal to *U*_eq_ of the major component. Pairs of S—C and C—C distances were restrained to within 0.01 Å of each other. The azomethine C═N unit is also disordered; the positions and anisotropic displacement parameters of the primed atoms were set to those of the unprimed ones. A short H···H contact (2.09 Å) involving the methyl groups of the disordered SEt residue and the DMSO molecule is noted. The final difference Fourier map had a peak at 0.91 Å from U and a hole at 0.11 Å from S1'. Owing to poor agreement, the (1 1 0) reflection was omitted from the final refinement.

### Crystal data

**Table:**

[U(C_17_H_15_N_3_O_2_S)O_2_(C_2_H_6_OS)]	*F*(000) = 1280
*M**_r_* = 673.54	*D*_x_ = 2.097 Mg m^−^^3^
Monoclinic, *P*2_1_/*n*	Mo *K*α radiation, λ = 0.71073 Å
Hall symbol: -P 2yn	Cell parameters from 8576 reflections
*a* = 11.6988 (3) Å	θ = 2.2–27.5°
*b* = 15.4972 (3) Å	µ = 7.84 mm^−^^1^
*c* = 12.2246 (3) Å	*T* = 100 K
β = 105.714 (3)°	Prism, red
*V* = 2133.47 (9) Å^3^	0.18 × 0.12 × 0.10 mm
*Z* = 4	

### Data collection

**Table:**

Agilent SuperNova Dual diffractometer with an Atlas detector	4927 independent reflections
Radiation source: SuperNova (Mo) X-ray Source	4237 reflections with *I* > 2σ(*I*)
Mirror monochromator	*R*_int_ = 0.044
Detector resolution: 10.4041 pixels mm^-1^	θ_max_ = 27.6°, θ_min_ = 2.5°
ω scan	*h* = −14→15
Absorption correction: multi-scan (*CrysAlis PRO*; Agilent, 2010)	*k* = −20→20
*T*_min_ = 0.333, *T*_max_ = 0.508	*l* = −15→11
19265 measured reflections	

### Refinement

**Table:**

Refinement on *F*^2^	Primary atom site location: structure-invariant direct methods
Least-squares matrix: full	Secondary atom site location: difference Fourier map
*R*[*F*^2^ > 2σ(*F*^2^)] = 0.030	Hydrogen site location: inferred from neighbouring sites
*wR*(*F*^2^) = 0.063	H-atom parameters constrained
*S* = 1.01	*w* = 1/[σ^2^(*F*_o_^2^) + (0.0243*P*)^2^ + 4.2587*P*] where *P* = (*F*_o_^2^ + 2*F*_c_^2^)/3
4927 reflections	(Δ/σ)_max_ = 0.001
281 parameters	Δρ_max_ = 0.98 e Å^−^^3^
3 restraints	Δρ_min_ = −1.52 e Å^−^^3^

### Fractional atomic coordinates and isotropic or equivalent isotropic displacement parameters (Å^2^)

**Table:**

	*x*	*y*	*z*	*U*_iso_*/*U*_eq_	Occ. (<1)
U	0.364161 (13)	0.640410 (10)	0.689855 (13)	0.01292 (6)	
S1	0.15547 (15)	0.46851 (11)	0.34195 (14)	0.0341 (5)	0.828 (3)
S1'	0.0795 (7)	0.3967 (5)	0.5070 (7)	0.034*	0.172 (3)
S2	0.58169 (11)	0.77489 (8)	0.88184 (11)	0.0260 (3)	
O1	0.4057 (3)	0.72624 (19)	0.5554 (3)	0.0171 (7)	
O2	0.3653 (3)	0.5871 (2)	0.8599 (3)	0.0257 (8)	
O3	0.2236 (3)	0.69271 (19)	0.6677 (3)	0.0169 (7)	
O4	0.5046 (2)	0.58833 (18)	0.7065 (3)	0.0168 (7)	
O5	0.4562 (3)	0.7596 (2)	0.8050 (3)	0.0220 (7)	
N1	0.3115 (3)	0.5629 (2)	0.4990 (3)	0.0145 (8)	
N2	0.1805 (3)	0.4702 (2)	0.5585 (3)	0.0211 (9)	0.828 (3)
C8'	0.1805 (3)	0.4702 (2)	0.5585 (3)	0.0211 (9)	0.172
N3	0.2299 (3)	0.5040 (2)	0.6674 (3)	0.0150 (8)	
C1	0.4713 (4)	0.7110 (3)	0.4855 (4)	0.0140 (9)	
C2	0.5515 (4)	0.7744 (3)	0.4682 (4)	0.0194 (10)	
H2	0.5603	0.8270	0.5096	0.023*	
C3	0.6170 (4)	0.7605 (3)	0.3915 (4)	0.0204 (10)	
H3	0.6708	0.8036	0.3812	0.025*	
C4	0.6058 (4)	0.6841 (3)	0.3285 (4)	0.0195 (10)	
H4	0.6517	0.6756	0.2761	0.023*	
C5	0.5290 (4)	0.6222 (3)	0.3427 (4)	0.0194 (10)	
H5	0.5202	0.5709	0.2986	0.023*	
C6	0.4613 (4)	0.6331 (3)	0.4226 (4)	0.0157 (9)	
C7	0.3764 (4)	0.5671 (3)	0.4269 (4)	0.0148 (9)	
H7	0.3662	0.5222	0.3721	0.018*	
N2'	0.2226 (4)	0.4984 (3)	0.4777 (4)	0.0225 (10)	0.172 (3)
C8	0.2226 (4)	0.4984 (3)	0.4777 (4)	0.0225 (10)	0.828 (3)
C9	0.0485 (5)	0.3874 (4)	0.3578 (6)	0.0248 (15)	0.828 (3)
H9A	0.0055	0.4093	0.4116	0.030*	0.828 (3)
H9B	−0.0105	0.3790	0.2834	0.030*	0.828 (3)
C9'	0.097 (3)	0.3909 (19)	0.3636 (16)	0.025*	0.172 (3)
H9'A	0.0164	0.3980	0.3113	0.030*	0.172 (3)
H9'B	0.1427	0.4426	0.3538	0.030*	0.172 (3)
C10	0.1023 (6)	0.3020 (5)	0.3993 (6)	0.0410 (18)	0.828 (3)
H10A	0.0395	0.2618	0.4051	0.061*	0.828 (3)
H10B	0.1588	0.3091	0.4742	0.061*	0.828 (3)
H10C	0.1437	0.2791	0.3458	0.061*	0.828 (3)
C10'	0.152 (3)	0.3162 (19)	0.320 (3)	0.041*	0.172 (3)
H10D	0.1517	0.3270	0.2410	0.061*	0.172 (3)
H10E	0.1065	0.2637	0.3240	0.061*	0.172 (3)
H10F	0.2339	0.3088	0.3665	0.061*	0.172 (3)
C11	0.1832 (4)	0.4694 (3)	0.7413 (4)	0.0167 (9)	
H11	0.1265	0.4251	0.7142	0.020*	
C12	0.2075 (4)	0.4904 (3)	0.8601 (4)	0.0183 (10)	
C13	0.1371 (4)	0.4507 (3)	0.9225 (4)	0.0241 (11)	
H13	0.0770	0.4111	0.8856	0.029*	
C14	0.1538 (4)	0.4683 (4)	1.0362 (4)	0.0290 (12)	
H14	0.1043	0.4424	1.0770	0.035*	
C15	0.2443 (5)	0.5245 (3)	1.0904 (4)	0.0274 (11)	
H15	0.2567	0.5363	1.1690	0.033*	
C16	0.3160 (4)	0.5633 (3)	1.0326 (4)	0.0247 (11)	
H16	0.3781	0.6004	1.0720	0.030*	
C17	0.2984 (4)	0.5486 (3)	0.9154 (4)	0.0185 (10)	
C18	0.6216 (5)	0.6806 (3)	0.9658 (4)	0.0290 (12)	
H18A	0.5758	0.6778	1.0219	0.044*	
H18B	0.7066	0.6824	1.0050	0.044*	
H18C	0.6046	0.6296	0.9168	0.044*	
C19	0.6776 (5)	0.7611 (5)	0.7925 (5)	0.0467 (16)	
H19A	0.6667	0.8091	0.7384	0.070*	
H19B	0.6589	0.7066	0.7508	0.070*	
H19C	0.7603	0.7599	0.8390	0.070*	

### Atomic displacement parameters (Å^2^)

**Table:**

	*U*^11^	*U*^22^	*U*^33^	*U*^12^	*U*^13^	*U*^23^
U	0.01231 (9)	0.01200 (8)	0.01301 (9)	−0.00072 (6)	0.00096 (6)	−0.00015 (7)
S1	0.0401 (10)	0.0365 (9)	0.0277 (9)	−0.0149 (8)	0.0128 (8)	−0.0043 (8)
S2	0.0302 (7)	0.0166 (6)	0.0228 (6)	−0.0051 (5)	−0.0073 (5)	−0.0024 (5)
O1	0.0220 (16)	0.0111 (14)	0.0189 (16)	−0.0001 (12)	0.0067 (14)	0.0000 (13)
O2	0.0299 (18)	0.0282 (18)	0.0153 (16)	−0.0136 (15)	−0.0002 (15)	0.0032 (15)
O3	0.0139 (15)	0.0169 (15)	0.0200 (16)	0.0015 (13)	0.0043 (13)	0.0003 (14)
O4	0.0142 (15)	0.0110 (14)	0.0210 (16)	−0.0005 (12)	−0.0025 (13)	−0.0033 (13)
O5	0.0246 (17)	0.0159 (16)	0.0203 (17)	0.0008 (13)	−0.0027 (15)	−0.0029 (14)
N1	0.0130 (17)	0.0141 (18)	0.0143 (18)	−0.0002 (14)	0.0000 (15)	0.0009 (16)
N2	0.024 (2)	0.021 (2)	0.015 (2)	−0.0050 (17)	−0.0008 (18)	−0.0024 (18)
C8'	0.024 (2)	0.021 (2)	0.015 (2)	−0.0050 (17)	−0.0008 (18)	−0.0024 (18)
N3	0.0130 (17)	0.0159 (18)	0.0138 (18)	−0.0002 (15)	0.0001 (15)	0.0008 (16)
C1	0.0110 (19)	0.015 (2)	0.014 (2)	0.0033 (17)	−0.0005 (18)	0.0018 (18)
C2	0.024 (2)	0.014 (2)	0.020 (2)	−0.0011 (18)	0.006 (2)	0.0013 (19)
C3	0.021 (2)	0.015 (2)	0.026 (3)	−0.0033 (18)	0.008 (2)	0.005 (2)
C4	0.020 (2)	0.021 (2)	0.019 (2)	0.0057 (19)	0.010 (2)	0.004 (2)
C5	0.020 (2)	0.018 (2)	0.022 (2)	0.0042 (18)	0.010 (2)	0.003 (2)
C6	0.016 (2)	0.014 (2)	0.015 (2)	0.0026 (17)	0.0004 (18)	0.0012 (18)
C7	0.017 (2)	0.013 (2)	0.012 (2)	−0.0002 (17)	0.0006 (18)	−0.0017 (18)
N2'	0.021 (2)	0.018 (2)	0.022 (2)	−0.0031 (19)	−0.007 (2)	0.003 (2)
C8	0.021 (2)	0.018 (2)	0.022 (2)	−0.0031 (19)	−0.007 (2)	0.003 (2)
C9	0.013 (3)	0.026 (3)	0.033 (4)	−0.008 (3)	0.000 (3)	−0.005 (3)
C10	0.035 (4)	0.046 (4)	0.041 (4)	−0.009 (3)	0.008 (3)	−0.001 (4)
C11	0.012 (2)	0.015 (2)	0.020 (2)	−0.0007 (17)	−0.0003 (19)	0.0026 (19)
C12	0.017 (2)	0.019 (2)	0.017 (2)	0.0047 (18)	0.0018 (19)	0.004 (2)
C13	0.020 (2)	0.028 (3)	0.024 (3)	0.001 (2)	0.004 (2)	0.002 (2)
C14	0.025 (3)	0.041 (3)	0.024 (3)	0.001 (2)	0.012 (2)	0.009 (2)
C15	0.037 (3)	0.030 (3)	0.017 (2)	0.013 (2)	0.009 (2)	0.003 (2)
C16	0.033 (3)	0.021 (2)	0.015 (2)	0.001 (2)	−0.002 (2)	0.001 (2)
C17	0.024 (2)	0.012 (2)	0.017 (2)	0.0016 (18)	0.001 (2)	0.0013 (19)
C18	0.032 (3)	0.019 (2)	0.026 (3)	0.002 (2)	−0.009 (2)	0.000 (2)
C19	0.034 (3)	0.066 (4)	0.036 (3)	−0.020 (3)	0.004 (3)	−0.007 (3)

### Geometric parameters (Å, º)

**Table:**

U—O1	2.267 (3)	C9—C10	1.494 (9)
U—O2	2.233 (3)	C9—H9A	0.9900
U—O3	1.787 (3)	C9—H9B	0.9900
U—O4	1.792 (3)	C9'—C10'	1.493 (13)
U—O5	2.395 (3)	C9'—H9'A	0.9900
U—N1	2.547 (4)	C9'—H9'B	0.9900
U—N3	2.603 (4)	C10—H10A	0.9800
S1—C9	1.821 (6)	C10—H10B	0.9800
S1'—C9'	1.819 (12)	C10—H10C	0.9800
S2—O5	1.532 (3)	C10'—H10D	0.9800
S2—C18	1.773 (5)	C10'—H10E	0.9800
S2—C19	1.779 (6)	C10'—H10F	0.9800
O1—C1	1.316 (5)	C11—C12	1.440 (6)
O2—C17	1.310 (6)	C11—H11	0.9500
N1—C7	1.312 (5)	C12—C13	1.407 (7)
N1—N2'	1.415 (5)	C12—C17	1.419 (6)
N2—N3	1.402 (5)	C13—C14	1.377 (7)
N3—C11	1.292 (6)	C13—H13	0.9500
C1—C2	1.414 (6)	C14—C15	1.393 (7)
C1—C6	1.419 (6)	C14—H14	0.9500
C2—C3	1.379 (6)	C15—C16	1.373 (7)
C2—H2	0.9500	C15—H15	0.9500
C3—C4	1.398 (6)	C16—C17	1.409 (6)
C3—H3	0.9500	C16—H16	0.9500
C4—C5	1.358 (6)	C18—H18A	0.9800
C4—H4	0.9500	C18—H18B	0.9800
C5—C6	1.425 (6)	C18—H18C	0.9800
C5—H5	0.9500	C19—H19A	0.9800
C6—C7	1.437 (6)	C19—H19B	0.9800
C7—H7	0.9500	C19—H19C	0.9800
			
O3—U—O4	177.84 (14)	S1—C9—H9A	108.7
O3—U—O2	94.71 (13)	C10—C9—H9B	108.7
O4—U—O2	87.36 (13)	S1—C9—H9B	108.7
O3—U—O1	89.60 (12)	H9A—C9—H9B	107.6
O4—U—O1	88.65 (12)	C10'—C9'—S1'	124 (2)
O2—U—O1	160.62 (11)	C10'—C9'—H9'A	106.4
O3—U—O5	89.31 (12)	S1'—C9'—H9'A	106.4
O4—U—O5	91.63 (12)	C10'—C9'—H9'B	106.4
O2—U—O5	81.36 (11)	S1'—C9'—H9'B	106.4
O1—U—O5	79.81 (11)	H9'A—C9'—H9'B	106.5
O3—U—N1	95.21 (13)	C9—C10—H10A	109.5
O4—U—N1	83.00 (12)	C9—C10—H10B	109.5
O2—U—N1	128.10 (12)	H10A—C10—H10B	109.5
O1—U—N1	70.05 (11)	C9—C10—H10C	109.5
O5—U—N1	149.45 (11)	H10A—C10—H10C	109.5
O3—U—N3	81.27 (12)	H10B—C10—H10C	109.5
O4—U—N3	98.90 (12)	C9'—C10'—H10D	109.5
O2—U—N3	69.45 (11)	C9'—C10'—H10E	109.5
O1—U—N3	129.92 (11)	H10D—C10'—H10E	109.5
O5—U—N3	148.30 (11)	C9'—C10'—H10F	109.5
N1—U—N3	62.04 (11)	H10D—C10'—H10F	109.5
O5—S2—C18	106.6 (2)	H10E—C10'—H10F	109.5
O5—S2—C19	105.3 (2)	N3—C11—C12	127.3 (4)
C18—S2—C19	98.3 (3)	N3—C11—H11	116.3
C1—O1—U	130.0 (3)	C12—C11—H11	116.3
C17—O2—U	142.8 (3)	C13—C12—C17	119.5 (4)
S2—O5—U	133.10 (18)	C13—C12—C11	117.6 (4)
C7—N1—N2'	116.1 (4)	C17—C12—C11	122.8 (4)
C7—N1—U	123.5 (3)	C14—C13—C12	121.2 (5)
N2'—N1—U	119.1 (3)	C14—C13—H13	119.4
C11—N3—N2	111.4 (4)	C12—C13—H13	119.4
C11—N3—U	128.4 (3)	C13—C14—C15	119.0 (5)
N2—N3—U	119.0 (3)	C13—C14—H14	120.5
O1—C1—C2	120.0 (4)	C15—C14—H14	120.5
O1—C1—C6	121.8 (4)	C16—C15—C14	121.4 (5)
C2—C1—C6	118.1 (4)	C16—C15—H15	119.3
C3—C2—C1	120.4 (4)	C14—C15—H15	119.3
C3—C2—H2	119.8	C15—C16—C17	120.8 (5)
C1—C2—H2	119.8	C15—C16—H16	119.6
C2—C3—C4	121.4 (4)	C17—C16—H16	119.6
C2—C3—H3	119.3	O2—C17—C16	120.7 (4)
C4—C3—H3	119.3	O2—C17—C12	121.2 (4)
C5—C4—C3	119.7 (4)	C16—C17—C12	118.1 (4)
C5—C4—H4	120.2	S2—C18—H18A	109.5
C3—C4—H4	120.2	S2—C18—H18B	109.5
C4—C5—C6	120.9 (4)	H18A—C18—H18B	109.5
C4—C5—H5	119.5	S2—C18—H18C	109.5
C6—C5—H5	119.5	H18A—C18—H18C	109.5
C5—C6—C1	119.5 (4)	H18B—C18—H18C	109.5
C5—C6—C7	117.3 (4)	S2—C19—H19A	109.5
C1—C6—C7	122.9 (4)	S2—C19—H19B	109.5
N1—C7—C6	126.2 (4)	H19A—C19—H19B	109.5
N1—C7—H7	116.9	S2—C19—H19C	109.5
C6—C7—H7	116.9	H19A—C19—H19C	109.5
C10—C9—S1	114.2 (4)	H19B—C19—H19C	109.5
C10—C9—H9A	108.7		
			
O3—U—O1—C1	−149.9 (3)	O4—U—N3—N2	−89.6 (3)
O4—U—O1—C1	28.9 (3)	O2—U—N3—N2	−173.4 (3)
O2—U—O1—C1	107.0 (4)	O1—U—N3—N2	6.1 (3)
O5—U—O1—C1	120.8 (3)	O5—U—N3—N2	162.5 (3)
N1—U—O1—C1	−54.2 (3)	N1—U—N3—N2	−12.5 (3)
N3—U—O1—C1	−71.6 (4)	U—O1—C1—C2	−135.9 (3)
O3—U—O2—C17	43.8 (5)	U—O1—C1—C6	46.5 (5)
O4—U—O2—C17	−135.5 (5)	O1—C1—C2—C3	−177.2 (4)
O1—U—O2—C17	146.2 (4)	C6—C1—C2—C3	0.5 (6)
O5—U—O2—C17	132.4 (5)	C1—C2—C3—C4	0.4 (7)
N1—U—O2—C17	−56.6 (5)	C2—C3—C4—C5	0.1 (7)
N3—U—O2—C17	−35.0 (5)	C3—C4—C5—C6	−1.4 (7)
C18—S2—O5—U	−46.1 (3)	C4—C5—C6—C1	2.2 (7)
C19—S2—O5—U	57.7 (3)	C4—C5—C6—C7	175.9 (4)
O3—U—O5—S2	170.9 (3)	O1—C1—C6—C5	175.9 (4)
O4—U—O5—S2	−11.0 (3)	C2—C1—C6—C5	−1.7 (6)
O2—U—O5—S2	76.1 (3)	O1—C1—C6—C7	2.6 (6)
O1—U—O5—S2	−99.3 (3)	C2—C1—C6—C7	−175.1 (4)
N1—U—O5—S2	−90.0 (3)	N2'—N1—C7—C6	171.9 (4)
N3—U—O5—S2	98.9 (3)	U—N1—C7—C6	−21.1 (6)
O3—U—N1—C7	127.2 (3)	C5—C6—C7—N1	175.5 (4)
O4—U—N1—C7	−51.6 (3)	C1—C6—C7—N1	−11.0 (7)
O2—U—N1—C7	−132.7 (3)	N2—N3—C11—C12	−177.0 (4)
O1—U—N1—C7	39.5 (3)	U—N3—C11—C12	−9.7 (6)
O5—U—N1—C7	29.7 (4)	N3—C11—C12—C13	173.4 (4)
N3—U—N1—C7	−155.6 (4)	N3—C11—C12—C17	−6.6 (7)
O3—U—N1—N2'	−66.2 (3)	C17—C12—C13—C14	0.9 (7)
O4—U—N1—N2'	115.1 (3)	C11—C12—C13—C14	−179.2 (4)
O2—U—N1—N2'	34.0 (3)	C12—C13—C14—C15	−1.8 (7)
O1—U—N1—N2'	−153.8 (3)	C13—C14—C15—C16	0.7 (8)
O5—U—N1—N2'	−163.7 (3)	C14—C15—C16—C17	1.3 (7)
N3—U—N1—N2'	11.1 (3)	U—O2—C17—C16	−148.3 (4)
O3—U—N3—C11	−78.3 (4)	U—O2—C17—C12	32.4 (7)
O4—U—N3—C11	103.9 (4)	C15—C16—C17—O2	178.5 (4)
O2—U—N3—C11	20.1 (3)	C15—C16—C17—C12	−2.2 (7)
O1—U—N3—C11	−160.4 (3)	C13—C12—C17—O2	−179.6 (4)
O5—U—N3—C11	−4.1 (5)	C11—C12—C17—O2	0.4 (7)
N1—U—N3—C11	−179.0 (4)	C13—C12—C17—C16	1.1 (6)
O3—U—N3—N2	88.2 (3)	C11—C12—C17—C16	−178.8 (4)

### Hydrogen-bond geometry (Å, º)

**Table:**

*D*—H···*A*	*D*—H	H···*A*	*D*···*A*	*D*—H···*A*
C5—H5···O4^i^	0.95	2.48	3.322 (5)	147

Symmetry code: (i) −*x*+1, −*y*+1, −*z*+1.
